# Draft Genome Sequence of *Ochrobactrum anthropi* Strain W13P3, a Halotolerant Polycyclic Aromatic Hydrocarbon-Degrading Bacterium

**DOI:** 10.1128/genomeA.00867-15

**Published:** 2015-07-30

**Authors:** Xinxin Wang, Decai Jin, Lisha Zhou, Zhuo Zhang

**Affiliations:** a School of Environmental Science and Engineering, Tianjin University, Tianjin, China; b China Offshore Environmental Service Co. Ltd., Tianjin, China; c Research Center for Eco-Environmental Sciences, Chinese Academy of Sciences, Beijing, China; d Shenzhen Key Laboratory of Environmental Microbial Genomics and Application, BGI, Shenzhen, China; e Hunan Plant Protection Institute, Changsha, China

## Abstract

*Ochrobactrum anthropi* W13P3 was isolated from saline soil contaminated by polycyclic aromatic hydrocarbons (PAHs) and could degrade PAHs with 5% NaCl. We report the 5.3-Mb draft genome sequence of this strain, which is helpful for understanding the diversity of *Ochrobactrum* spp. and the mechanism of PAH degradation in saline environments.

## GENOME ANNOUNCEMENT

Polycyclic aromatic hydrocarbons (PAHs) are widespread hazardous contaminants that can be degraded by a number of bacteria, such as members of the genus *Ochrobactrum* (1–3). However, genomic information about PAH-degrading *Ochrobactrum* is still limited. The draft genome sequence of halotolerant PAH-degrading *Ochrobactrum anthropi* W13P3 is reported for the first time, isolated from a PAH-contaminated saline site (4).

Genomic DNA was extracted and sequenced by Illumina HiSeq 2000, which produced 14,006,606 paired-end reads with about 260-fold coverage. Reads were filtered, assembled, scaffolded, gap filled, and validated by the NGS QC toolkit version 2.3 (5), SOAPdenovo version 2.04 (6), SSPACE version 2.0 (7), GapFiller version 1.10 (8), and the Burrows-Wheeler alignment tool version 0.7.4 (9). This assembly generated 48 contigs with an *N*_50_ length of 475,065 bp and an average length of 109,953 bp, which were assembled into 43 scaffolds with an *N*_50_ length of 475,065 bp and an average length of 122,740 bp. The genome sequence was annotated by the NCBI Prokaryotic Genome Automatic Annotation Pipeline (PGAAP) (http://www.ncbi.nlm.nih.gov/genome/annotation_prok).

The draft genome comprised 5.3 Mb with a GC content of 56.3%. A total of 4,965 coding sequences (CDSs), 53 tRNA genes, 1 noncoding RNA (ncRNA), 1 rRNA operon, and 53 pseudogenes were identified. Of the CDSs, 50.1% can be assigned into 2,029 KEGG orthologous groups by KAAS (10), involving 182 metabolic pathways, and 72.1% can be assigned to COGs with amino acid transport and metabolism as the most abundant class. ISFinder revealed that the IS3 family dominated the insertion sequence (IS) elements (11). A total of 62 tandem repeats were detected by Tandem repeats finder version 4.07 (12), and 451 potentially secreted proteins were identified by SignalP version 4.0 (13). The plasmid stabilization gene *Par*B was detected on contig 17, which suggests the occurrence of plasmid. One incomplete prophage sequence was identified by PHAST (14). A clustered regularly interspaced short palindromic repeat (CRISPR) element was not detected by CRISPRFinder (15). Average nucleotide identity (ANI) analysis (16) revealed that *O. anthropi* W13P3 is phylogenetically related to *O. anthropi* ATCC 49188 (97.0%) (17), *O. anthropi* CTS325 (98.8%) (18), *O. anthropi* ML7 (98.9%) (19), and *O. anthropi* ATCC 49687 (97.1%) (20).

Two protocatechuate 3,4-dioxygenase genes and 3 alkane 1-monooxygenase genes were identified, which were responsible for the degradation of PAHs and alkanes. Moreover, 8 genes were identified as being involved in the uptake and synthesis of compatible solute, including 6 glycine/betaine ABC transporter genes, 1 ectoine synthase gene, and 1 betaine-aldehyde dehydrogenase gene. Nitrogen fixation genes were detected, which were responsible for the assimilation of gaseous nitrogen. These genes may be essential to survival in a saline oligotrophic environment. Information about the genome sequence of *O. anthropi* W13P3 offered an opportunity to understand the genetic diversity of *Ochrobactrum* spp. and the mechanism of PAH degradation in saline environments.

### Nucleotide sequence accession number.

The draft genome sequence of *O. anthropi* W13P3 was deposited in GenBank under the accession number JENZ00000000. The version described in this paper is the first version.
